# An Open Letter on Advancing HIV prevention: Augmenting an ecosystem-based approach to understand prevention decision-making

**DOI:** 10.12688/gatesopenres.16067.1

**Published:** 2024-08-02

**Authors:** Nishan Gantayat, James Baer, Alok Gangaramany, Rosemary Pierce-Messick

**Affiliations:** 1 Final Mile Consulting, New York, New York, 10007, USA; 2 Independent Consultant, London, UK

**Keywords:** HIV Prevention, Public Health, HIV/AIDS, Ecosystem, Decision-making, Behavioral Science

## Abstract

In the last two decades, HIV programs have been able to avert millions of AIDS-related deaths and reduce HIV incidence. However, the 1.3 million new HIV infections in 2022 remain significantly above the UNAIDS target of fewer than 370,000 new infections by 2025. HIV programs worldwide also did not achieve the UN’s 90-90-90 target for testing and treatment set for 2020. Within this broader picture, HIV continues to disproportionately affect key and at-risk populations, including gay men and other men who have sex with men, female sex workers, and adolescent girls and young women. As HIV incidence declines and biomedical advances continue, it will become critical for public-health practitioners to reach key and at-risk populations with prevention services and limit primary transmission.

In this Open Letter, we focus on demand for HIV prevention to illuminate factors that influence uptake of HIV prevention products and services. These factors exist at three levels of the decision-making ecosystem – the individual level, interaction level and systemic level. We argue that approaching HIV prevention solely through the lens of these levels creates a static view of prevention decision-making. There is a need instead for a dynamic viewpoint that can mirror the changing contexts in which users find themselves and make prevention decisions. We demonstrate that the current ecosystem viewpoint is useful to understand the gaps that exist in program implementation, but does not provide adequate insights into the underlying behaviors that contribute to these gaps. To address this, we suggest an approach to include dynamic aspects of decision-making with factors that influence the individual’s assessment of risk, their evaluation of the opportunities to use HIV prevention, and their effective use of prevention products.

## Introduction

There were around 1.3 million new cases of HIV infection in 2022, marking a 59% decline from the peak of new infections in 1995 ^1^. Five countries have already achieved the United Nations 95-95-95 targets set for 2025 (95% of those who are infected with HIV are diagnosed, 95% of those diagnosed are on ART, and 95% of those on ART have untransmissible levels of HIV), which are integral to the UNAIDS strategy to eliminate HIV as a public health threat by 2030 ^2^. Among those infected with HIV, approximately 20.8 million AIDS-related deaths have been averted between 1996 and 2022 due to antiretroviral therapy (ART) ^3^.

The success of HIV programs over the years underscores the importance of strong commitments to public health, including evidence-based policymaking, scaling up of services for those living with HIV, and an increased focus on prevention programs. Advances in biomedical products now present individuals with a range of HIV prevention choices, and the introduction of the dapivirine vaginal ring for women and LA-CAB (long-acting injectable ART) is expected to greatly support the effort to reduce HIV infections ^4^.

However, the 1.3 million annual new HIV infections remain significantly above the UNAIDS target of fewer than 370,000 new infections by 2025 ^5^. Overall, HIV programs worldwide also did not achieve the UN’s 90-90-90 target of testing and treatment set for 2020 ^6^. Even though new infections have dropped by more than 40% among adolescent girls and boys, adolescent girls and young women remain an at-risk population, with 4,000 acquiring HIV every week ^1^. Yet in sub-Saharan Africa, only 42% of districts with very high HIV incidence have dedicated prevention programs for adolescent girls and young women ^1^.

The inequities are amplified for key populations, including sex workers, men who have sex with men, transgender people, and people who inject drugs. In a gap analysis for combination prevention in the period 2016–2020, sex workers and men who have sex with men showed significant gaps against targets for condom use at last high-risk sex, for HIV prevention program coverage, and for sexually transmitted infection (STI) screening in the last 3 months ^7^. In addition, laws that criminalize people from key populations or their behaviors remain on statute books across much of the world. In 168 countries, some aspect of sex work is criminalized; 67 countries criminalize consensual same-sex intercourse; 20 countries criminalize transgender people; and 143 countries criminalize or otherwise prosecute HIV exposure, non-disclosure, or transmission ^3^.

With limited access to or scarcity of HIV and other health services, and legal and social discrimination, the HIV pandemic continues to disproportionately impact key populations. In 2022, HIV prevalence was 11 times higher among gay men and other men who have sex with men, four times higher among sex workers, seven times higher among people who inject drugs, and 14 times higher among transgender people, compared with individuals from the general population ^3^. The overall success of reaching HIV prevention targets is therefore dependent on effective uptake of prevention products by key and at-risk populations.

Different from HIV treatment, HIV prevention comprises an array of interventions with varying frequency of use, and which are adopted in the absence of any immediate health challenges. This presents program planners, policymakers, and funding bodies with the challenge of driving effective prevention programs aligned with the user journey.

In this Open Letter, we use an ecosystem lens to illuminate factors that influence uptake of HIV prevention products and services. We consider three levels of the decision-making ecosystem. The individual level includes cognitive-psychological-behavioral factors such as relevance, risk saliency, coping ability, and control. The interaction level refers to the individual’s interactions with people and programs when engaging with HIV prevention services. Finally, the systemic level refers to structural factors that shape the individual’s ecosystem: these include culture, political institutions, programs, and policies. These factors may be barriers or enablers to the individual’s decision to use HIV prevention products. We consider the three levels with a focus on the HIV prevention landscape in southern and eastern Africa as it affects three key and at-risk populations: men who have sex with men, female sex workers, and adolescent girls and young women.

We further discuss that there is scope for augmentation of the current ecosystem approach to include the dynamic aspect of decision-making that usually accompanies uptake of HIV prevention products and services. We believe that HIV prevention will benefit from a more dynamic understanding of the factors that influence the individual’s assessment of risk, their evaluation of the opportunities to use HIV prevention, and their effective use of prevention products. Uptake of HIV prevention products and services should not be seen as a single behavior occurring at one time, but as the culmination of multiple behaviors and factors that often exist concurrently.

## Individual level

An individual’s actions related to HIV prevention are strongly influenced by their emotions, which influence the decisions they make, how they make them, and how they perceive and internalize the prevailing risks. This emotional process, explained further below and summarized in Table 1, includes aspects such as the relevance of HIV to them, their perception of risk, their ability to cope with the internalized risk, and control over their actions. Table 1 below summarizes the factors followed by a more detailed explanation.

**Table 1.** Summary table for individual level of the user ecosystem.

Individual level is centered on the end-user and includes the cognitive-psychological-behavioral factors affecting their HIV prevention decisions.	
**Relevance** *includes evaluation of importance of HIV prevention for a user*	Relevance for HIV prevention is often seen to be low as users such as adolescent girls and young women and men who have sex with men place higher priorities on economic benefits, safety and relationship stability as compared to HIV prevention.
**Risk saliency** *includes user’s attention towards immediate risks associated with HIV*	Risk saliency is affected due to absence of caution among peers, gender-based violence, lack of any symptoms associated with risk of HIV, and present bias towards immediate benefits of risky behaviors.
**Coping ability** *includes user’s ability to deal optimally with negative consequences of HIV prevention*	User requires ability to deal with stigma, fear of judgement and shame, anticipated disruption of normal life routines in pursuing prevention, side-effects, and privacy violations
Control *includes the ability of the user to use prevention products*	Control over prevention decisions related to HIV prevention is limited by low negotiation power in relationships in the case of adolescent girls and young women, anticipated violence for adolescent girls and young women and female sex workers, mental health and inebriation.

### Relevance

Habits surrounding HIV prevention, such as finding an appropriate prevention method, or being aware of vulnerabilities that make one susceptible to HIV, are seldom consistent or widespread among at-risk populations. One of the factors influencing this is the relevance of HIV prevention to the individual. HIV prevention has been observed to be a low priority among adolescent girls and young women, and among men who have sex with men, who may instead prioritize economic needs and opportunities, safety from violence, and managing their familial and social relationships ^8, 9^. Furthermore, a dominant mental model that one seeks medical help only when sick limits the relevance of prevention preparedness, as studies from Malawi and Rwanda among female sex workers and men who have sex with men have shown ^10, 11^. Strategies to make HIV prevention relevant include integrating HIV prevention services into other health services ^12^, and showing how HIV prevention serves other life goals: for example, women who have higher-order, long-term goals to become pregnant have been found more likely to adhere to oral pre-exposure prophylaxis (PrEP) ^13^.

### Risk saliency

Prevention action is also impacted by limited risk saliency, meaning that preventing HIV may not be top-of-mind when people are making decisions about sexual behavior (for example, because a potential partner shows no symptoms of HIV, or the individual believes that HIV is a manageable disease, or they have more pressing priorities than HIV prevention). Respondents in studies in South Africa, Kenya and Nigeria were optimistic that they would not get infected with HIV, and continued to engage in risky sexual behavior ^14– 16^. Limited risk saliency is also influenced by present bias – the tendency to prioritize perceived short-term gains at the cost of long-term outcomes ^17^.

Social norms also contribute to a suboptimal risk assessment. Young people find little or low concern for safety around HIV among their peers, and this can negatively affect their own risk perception ^18^. Particularly among men, stereotypes surrounding masculinity drive emotional suppression that can hinder information-seeking and support, thus affecting adequate assessment of HIV risks ^19^. For adolescent girls and young women and female sex workers, harmful gender norms lead to a discounting of the risk originating from gender-based violence, even though this places individuals at higher risk of HIV ^20^.

Another factor is that individuals generally take in HIV messages at times when they are not engaging in sexual behavior. In this “cold” state, deliberation on risks and benefits is optimally rational. But settings where unsafe risky behaviors take place often do not allow for optimal rational evaluation of risks and benefits. In this emotionally activated “hot” state, heuristics (mental short-cuts) are used for risk assessment. HIV prevention programs for adolescent girls and young women and men who have sex with men generally take place in out-of-context and safe (i.e. “cold state”) settings ^17^, but this may not equip them to perceive and accurately evaluate HIV risk at the time of sexual contact.

### Coping ability

Individuals may experience discomfort, fear and anxiety while exploring HIV prevention options. An inability to cope with these negative emotions impacts the uptake of prevention products and services. Fear of stigmatization is a significant barrier. In sub-Saharan Africa, fear of disclosing test results was driven by normative expectations of social ostracization and partner abandonment, leading to avoidance of healthcare facilities and affecting uptake of HIV testing ^21– 24^. These fears are disproportionately experienced by key populations ^25, 26^.

Similar reactions have also been associated with PrEP, which is often conflated in popular perception with ART, meaning that a person who is taking PrEP for HIV prevention may be mistakenly perceived as already infected with HIV. Men who have sex with men and young people, including adolescent girls and young women in Uganda, Zimbabwe, and South Africa, often encounter negative attitudes when seen to be using PrEP and are subjected to stigmatization ^27, 28^. For female sex workers, using HIV prevention products can signal that they are sex workers, leading to potential police harassment and social ostracization ^29^. A review of studies found that adolescent girls and young women’s uptake of condoms is affected by a fear that if they ask their partners to use a condom, they will themselves be judged as promiscuous and unfaithful ^26^.

Fear of disruption in normal life and the perceived impact upon one’s sex life affect an individual’s decisions around prevention. In South Africa, Nigeria, Kenya, and Zambia, condom use was associated with loss of pleasure due to the absence of “skin-to-skin” contact during protected sex ^30– 32^.

Fear of side-effects is a critical influencing factor. Pregnant women, men who have sex with men and transgender women in South Africa most frequently cited side-effects as the reason for stopping their use of PrEP during a one-year course, even though the side-effects monitored in the study were small ^33– 35^. In a qualitative study of adolescent girls and young women in Uganda, side-effects were also the main reason reported for stopping PrEP after 6 months of use ^36^. For the dapivirine vaginal ring (DVR, a silicone ring inserted in the vagina that releases an antiretroviral drug over a four-week period), perceived ring interference with sex, menstrual cycles, or potential harm to the vaginal environment have been expressed as concerns ^37^. Coping potential is also inadequate when it comes to anticipated pain. Corroborating a meta-analysis of the uptake of CAB-LA (a long-lasting injectable HIV prevention drug), a study conducted in Cape Town found that fear of the pain of injections impacted treatment uptake ^38, 39^.

mHealth interventions in Ghana and South Africa, have helped reduce the stigma and anticipated discrimination associated with in-person testing, and signing confidentiality agreements has helped enhance patient satisfaction and trust within clinics ^40, 41^. The confidentiality of HIV self-testing (HIVST), and control over PrEP use, are further enablers that combat fear of judgement. HIVST alleviates issues around lack of privacy and trustworthiness of providers ^42^. The provision of channels for HIVST other than physical stores has also shown success at improving self-testing, such as the rollout of self-screening at Central Chronic Medicines Dispensing and Distribution points (CCMDD) in South Africa ^43^. Community-focused interventions have helped promote effective use of PrEP, condoms, and HIV testing. These primarily seek to raise community awareness to help reduce stigma, along with peer support and educators who help the individual conduct HIVST or cope with taking PrEP ^44– 46^.

DVR could help address barriers to adherence seen with oral PrEP since it does not require regular daily uptake thus reducing fear of stigma ^47^. Early trials have found that DVR has been well tolerated by women, side-effects are low, and adherence and reported desire for continued use is high ^48– 50^. When given a choice between oral PrEP and DVR, two-thirds of adolescent girls and young women in the REACH program in South Africa, Zimbabwe, and Uganda chose DVR ^51^.

### Control

Though there has been considerable success in improving the range of choice when it comes to HIV prevention, exercising effective choice has not progressed at the same pace. Lack of individual agency, a sense of control, to negotiate use of HIV prevention products and services remains a significant barrier. Agency is affected by factors such as gender norms, violence, and mental health.

Women may be at risk of violence from sexual partners and have little power to negotiate the use of condoms. Female sex workers in South Africa and Ethiopia report a tradeoff between asking a client to use a condom during sex and remaining safe from violence from the client, in which they will prioritize their immediate safety ^52– 54^. Adolescent girls and young women across sub-Saharan Africa who may face violence or be unable to safely ensure condom use during sex have therefore expressed greater interest in PrEP to protect themselves from HIV ^55, 56^. Engaging partners in HIV prevention can improve risk evaluation within relationships. In the case of stable older relationships, partner engagement has improved last reported use of condom and led to a reduction in self-reported violence ^57^.

Poor mental health or inebriation may also reduce the ability to engage in prevention decision-making. Depression has been associated with lower adherence to PrEP and to ART ^58^. Women in East Africa, South Africa, Kenya, and Uganda who were depressed had lower adherence to PrEP ^59, 60^, but depression did not impact PrEP adherence among men in East Africa or women in Uganda ^58, 59^.

## Interaction level

The decision to take up HIV prevention products and services is made via one or more interactions (summarized in Table 2) with other people. These may occur during demand mobilization campaigns, partner/support system engagement programs, peer outreach, or while receiving HIV prevention services at health facilities. Whether these interactions address their specific needs, involve supportive providers, and engage other people in the individual’s ecosystem, will shape the individual’s prevention decisions.

**Table 2.** Summary table for interaction level of the user ecosystem.

*Interaction level includes the various interactions – with family and friends, health system, access points, media and communications, etc. – that a user is involved in while making the decision or action to undertake prevention*	
**Catering to multiple needs** *includes meeting an array of needs of users not limited to HIV but that directly or indirectly influence HIV prevention*	Users harbor multiple needs manifesting from different identities they associate themselves with, that go beyond HIV prevention and which are often not considered, resulting in inappropriate prevention-related messaging, need for delivery interventions such as DSD, service integration etc.
**Availability of supportive** stakeholders includes *critical influencers in a user’s ecosystem affecting HIV prevention decisions*	Users need/search for support from their close ties (family, peers) and service providers during uptake of prevention services and fear abandonment, judgement, or/and ostracization.
**Building engaged interactions** *includes promoting active participation of partners, family and peers in user’s prevention decision and journey*	User’s ecosystem needs to be constantly and meaningfully engaged, be it peers, partners, or family. Enhanced outreach programs, partner engagement during antenatal visits, partner-focused programs such as counselling have shown to positively influence uptake of HIV prevention.

### Catering to multiple needs

Interactions involving communication campaigns, and service delivery by providers, face several challenges, one of which is that at-risk populations are often subjected to a blanket approach, be it in product promotion or behavior change communications. This leads to non-alignment of HIV prevention with the precise identities and needs of users. For instance, messaging aimed broadly at men who have sex with men might not resonate with men who identify specifically as gay, and communication targeting an adolescent girl might not be relevant to a young woman ^61^. This mismatch between identities and communication content can increase avoidance of product communication messages and further isolate individuals from seeking appropriate HIV prevention opportunities. Communication on HIV prevention and risk also often does not consider how identities can shift within the same individual, e.g. HIV-negative to HIV-positive, or from not being pregnant to being pregnant ^62^. Each of these identities carry with it a different set of needs and vulnerabilities, which if not addressed or considered can lead to individuals being unable to appreciate their level of risk.

Interactions between user and providers are also hampered by service-related limitations such as product availability, facility hours, and staff shortages. A notable barrier at this level is low product availability. As of 2020, there was a shortage of 3.3 billion condoms distributed in eastern and southern Africa ^63^, and PrEP was only widely available in five countries’ national public health systems in the sub-region ^64^. In South Africa, provincial-level departments of health experience frequent shortages in PrEP supplies, which is a substantial barrier for uptake among pregnant and postpartum women in Cape Town ^34, 65^. A lack of testing kits has also been seen across sub-Saharan Africa ^23^.

Key populations have varying needs and barriers when it comes to accessing and using prevention methods such as PrEP, condoms, and HIV testing, and current service delivery models may therefore not work for all of them. Differentiated service delivery (DSD) has been successful in adapting service delivery to the differentiated needs and vulnerabilities of the individuals being served, such as through fast-tracked collection of HIV medication, community pick-up points or home delivery, multi-month dispensing and frequent refill options, as well as adherence support groups and extended clinic hours ^66– 70^. DSD has primarily been used to promote ART adherence, but there is a growing focus on the need and opportunity to expand this to other prevention services such as PrEP and HIV testing where barriers to access remain pressing ^71^.

A recent shift in DSD is toward integration of services ^12, 72, 73^. HIV can be integrated with services for non-communicable diseases and TB, as well as for STI testing, family planning, and gender-affirming hormone therapy ^74, 75^. For example, PrEP is being integrated into family planning services, antenatal clinics, and post-abortion care services in Kenya ^74^. Other health services can also be integrated into existing HIV services, such as offering hypertension and diabetes management at ART adherence clubs ^76^. There is an opportunity for greater integration of HIV services and mental health, especially given that vulnerabilities such as depression and substance use impact HIV prevention adherence, although there is currently little evidence of integration in this area.

Service integration as part of policy, as well as program design, can support both DSD as well as combination prevention, i.e. the combining of biomedical, behavioral, and structural interventions ^77^. This may be especially important for improving service delivery for key populations, as they face complex and varied barriers such as legal limitations, social stigma and provider bias, gender-based violence and social and economic inequalities ^77, 78^. An analysis of studies to promote condom use among female sex workers in sub-Saharan Africa found that combination prevention was highly effective. Effective interventions included educating female sex workers and creating supportive work environments, while others involved peer support combined with free condoms provided through peers as well as health providers ^79^.

### Availability of supportive stakeholders

Health systems and programs have found it difficult to involve crucial stakeholders like family and religious leaders in HIV communication efforts because of prevailing social norms stigmatizing key populations. The inability to appeal to the strong ties of family and religion often prevents programs and providers from leveraging credible, trustworthy sources of information and support in outreach to key populations.

Healthcare providers are often biased against key populations and are sometimes ignorant of their obligation to maintain the confidentiality of their patients ^80^. A study in Ghana found providers preaching religious teachings to men who have sex with men seeking treatment, and sometimes outright ignoring them ^81^. Negative attitudes among healthcare providers in South Africa hinder the promotion and delivery of PrEP to young people ^82^. Other studies in sub-Saharan Africa have also highlighted how provider bias obstructs treatment access for young people, including adolescent girls and young women, who are subjected to unnecessary and demeaning conditions, impeding their reproductive health choices ^83– 85^.

One way to enable more open and stigma-free interactions between key populations and healthcare providers is sensitivity training to help providers better understand the behaviors and needs of key populations ^74^. Kenya and Zimbabwe’s national policies suggest the use of the “train the trainer” model, in which healthcare providers train other providers, creating a snowball effect and speeding up the de-stigmatization of healthcare settings ^86^. In Kenya, men who have sex with men who are HIV+ being engaged through culturally trained peer educators have improved willingness to be engaged in treatment ^87^. Studies in Tanzania and Uganda showed that uptake of HIV prevention strategies rose to almost 70% when endorsed by religious leaders ^88, 89^. Support groups and peer educators and mentors have been seen to improve PrEP adherence for female sex workers, adolescent girls and young women, and men who have sex with men in Kenya ^64, 90^.

### Building engaged interactions

One approach to engaging individuals about HIV prevention is to involve key stakeholders within their ecosystem, including peers, partners, support groups, etc. The “enhanced peer outreach approach” capitalizes on key population individuals’ higher trust in their peers, leading to better engagement with HIV awareness programs, promoting safer sexual practices and increasing the probability that the individual attains the desired risk perception ^91– 93^. However, only limited studies have been done to show if peer led outreach impacts risk perception enough to alter risky sexual behaviors.

Women visiting antenatal and postpartum care in Kenya, Malawi, and Uganda have been given HIVST kits to share with their partners, which has improved testing uptake among these men ^94, 95^. Community-led monitoring and peer support has also shown to promote HIVST ^46^.

Another ecosystem-rooted engagement approach aims to engage men/partners in addressing vulnerabilities resulting from partner violence and harmful gender norms. Inequitable gender norms negatively impact women’s access to care, levels of physical and sexual violence and condom use. In stable relationships among older couples, male-partner engagement has improved the reported use of a condom on the most recent occasion of sex, and led to a reduction in self-reported violence ^57^. In Kenya, the “better family” intervention using couples counselling on HIV testing and care showed positive couple communication and efficacy to act around HIV ^96^. For adolescent girls and young women, school-based programs on HIV and violence reduction have shown positive results in terms of reduced acceptability to partner violence and decrease in multiple partners ^97^.

Support groups and peer educators and mentors have improved PrEP adherence for female sex workers, adolescent girls and young women, and men who have sex with men in Kenya ^64, 90^. DSD interventions that focus on flexible adherence support and enable more frequent visits may be particularly beneficial for adolescent girls and young women ^98^. There is also the potential to integrate social media into these services, such as the use of WhatsApp© chat groups for adherence clubs ^99^.

It can be important to differentiate between the communication preferences of different groups. A study in Nigeria showed that that female sex workers preferred face-to-face interactions, phone calls, and SMS for information regarding PrEP and HIVST, while men who have sex with men preferred digital platforms like WhatsApp, Facebook©, Instagram©, and Twitter© ^100^.

## Systemic level

In addition to the individual interactional levels, where the individual is firmly involved and holds a central place, there is a systemic level surrounding the individual. This is the environment in which individuals make prevention decisions and interact with others for prevention-related products and services. Four key factors ( Table 3) at this level impacting HIV-prevention behaviors are legal barriers, societal constraints involving dominant social norms, economic vulnerabilities, and education.

**Table 3.** Summary table for systemic level of the user ecosystem.

*Systemic level includes structural factors that significantly shape the ecosystem and include culture, political institutions, programs, and policies.*	
**Legal barriers** *include laws and policies enacted in a country that influence uptake of HIV prevention*	Legal barriers to HIV prevention consist of criminalization of same-sex marriage, LGBTQ people, and sex workers. Additionally, policies surrounding legal consent for adolescents, and lack of law enforcement protocols for gender and sexual violence, impede uptake of HIV prevention and hamper engagement with at-risk populations.
**Societal constraints** *include cultural and social beliefs surrounding gender, sexual behaviors, and HIV.*	HIV prevention is negatively impacted by harmful gender norms, stigma that often perpetrate fear and judgement, and intimate partner violence.
**Economic vulnerabilities** *include the disadvantageous financial and material circumstances of users.*	Economic difficulties arise due to unemployment, poverty, and gender inequity, resulting in lower uptake of prevention products, participation in HIV prevention programs, and prioritization of other needs over HIV prevention.
**Education** *includes factors associated with educational attainment and elements of schooling relevant for HIV prevention*	Education is a key determinant for HIV awareness and HIV prevention and is driven by the proportion of the population (girls in particular) enrolled in primary schools, the presence or absence of a comprehensive sexual education curriculum, school continuity, and risk-literacy programs/policies

### Legal barriers

Structural barriers lead to inadequate access to healthcare services for key populations, and insensitive service delivery. Laws criminalizing the identity or behaviors of key populations constitute a key barrier ^87^. A constraining legal environment leads to fear of – and actual – violence and harassment. This is evident for example in countries like Cameroon, Uganda, Nigeria, and Malawi, where criminalization limits the involvement of key populations in HIV prevention campaigns and access ^101– 104^. Criminalizing sex work obstructs HIV prevention efforts, subjects sex workers to police harassment and sexual violence ^105, 106^, and undermines safe-sex negotiations with clients ^107^. Similarly, laws penalizing same-sex relationships contribute to stigma and discrimination, and hinder HIV prevention interventions, perpetuating the epidemic among men who have sex with men ^108^. Countries that have decriminalized same-sex relations, such as South Africa and Botswana, have seen greater rates of testing and access to treatment among men who have sex with men ^78^.

Sex workers in China and Uganda must avoid accepting condoms from health services due to concerns that condoms will be used as evidence of sex workers’ occupation, which is illegal and can lead to imprisonment or even the death penalty ^109, 110^. Studies conducted in Ethiopia, Kenya, China, and India found evidence of violence by law enforcement against sex workers, with police demanding condomless sex. However, most of the evidence towards working on mitigating violence from law enforcement has been concentrated in community-led interventions and mobilization of female sex workers and men who have sex with men to take legal action ^106^. There has been limited evidence of state-level policy interventions acknowledging the existence of violence and prohibiting its perpetration on female sex workers and men who have sex with men ^111– 113^.

Strict consent laws for medical services in countries like Uganda and Kenya make it difficult for adolescents to access prevention until they are 18. This is particularly worrying for adolescent girls and young women at heightened risk of HIV, since the only way to access treatment is through explicit consent from their parents or guardians, which can lead to violence and social ostracization ^114^. However, several countries are making strides in addressing consent laws. South Africa’s reduction in the age of consent for medical services to 12 years saw significant increases in uptake of PrEP and HIV testing among teenagers ^115^. Tanzania also achieved comparable results after lowering the age to 12 ^114^.

### Societal constraints

Harmful gender norms make it difficult, especially for women, to exercise choice and agency to assess and access relevant HIV prevention opportunities. In Kenya, women who anticipate male-partner stigma or violence are more than twice as likely to refuse HIV testing ^116^. In Uganda, some women’s decision not to initiate PrEP was due to anticipated violence from their partners ^117, 118^. A study conducted in South Africa found that intimate partner violence and fear this can result in postpartum women not feeling confident enough to request condom use ^119^. Female sex workers who have been recent victims of violence find it even more difficult to use condoms with their male intimate partners than with their clients ^120^.

It has also been reported that men who endorse inequitable gender norms are less likely to take up HIV prevention products like condoms, or services like self-testing ^121^, as they fear it may be perceived as a sign of weakness or vulnerability ^122^. Prevailing gender norms, which may cause healthcare providers to hold negative attitudes, often discourage at-risk populations from openly discussing sexual health concerns and accessing appropriate care, as seen in studies from Malawi and Mozambique ^96, 123^. Gender norms that drive inequity contribute to a lack of agency for sex workers in negotiating condom use or asserting their sexual health needs ^124^. In South Africa, the sex worker organizations SWEAT and Sisonke run a national 24-hour toll-free helpline staffed by trained sex worker counsellors. The helpline addresses concerns including violence and police abuse, offering telephonic and face-to-face counselling. Referrals to paralegals and partner organizations are provided when needed ^106^.

### Economic vulnerabilities

Poverty and unemployment make at-risk populations, particularly women, vulnerable to health challenges due to increased risk-taking behavior. In the face of economic needs, many women resort to transactional sex to sustain their livelihoods, and young girls are often coerced into sexual activities with older men to survive. Studies across South Africa, Kenya, Malawi, and Uganda showed that people with a low socio-economic status (SES) reported significantly lower HIV testing uptake as well as lower usage of condoms than those with a high SES ^125– 127^. Low economic status has been associated with earlier first sexual experience, lower condom use during most recent sex, having multiple sex partners, increased chances that the first sex act is non-consensual, and a greater likelihood of having had transactional sex or physically forced sex ^128^. Programs with an economic-support component or cash-transfer schemes have been seen to lower risky sexual activity ^128^. Conditional cash-transfer programs like the *zomba* cash experiment in Malawi boosted the acceptability of HIV prevention products among adolescent girls and young women ^129^. In Tanzania, high-value cash transfers lowered STI prevalence through increased prevention product uptake ^130^.

Reduced funding, mostly for condoms and VMMC, along with challenges within health systems, including inefficient supply chain design for HIV products in southern and eastern Africa, limits programs’ ability to effectively reach and promote HIV prevention among key populations ^131, 132^. The efficiency of healthcare delivery systems also structurally impacts the availability and coverage of HIV prevention and treatment products. In Ethiopia, for example, poor supply chain management has led to stock-outs as well as poor data-keeping, and high rates of wastage ^133^. Uganda mitigated similar problems through training and on-site mentorship for its logistics management information systems, which reduced stock outages and improved record-keeping ^134^. Programmatic investments such as EpiC (Meeting Targets and Maintaining Epidemic Control) and BioPIC (Biomedical Prevention Implementation Collaborative) aim to improve program implementation and management, and health information systems through collaboration with local partners.

### Education

Education is a key structural determinant for awareness of HIV and demand mobilization. Increased education and higher levels of educational attainment have been shown to be linked to reduced HIV incidence and prevalence. In Botswana, each year of schooling has been associated with an 8.1 percentage-point drop in HIV incidence ^135^. Similarly, in Zimbabwe, among 15–18-year-old girls, those who are enrolled in school are more than five times less likely to have HIV than those who have dropped out ^136^. Though enrollment into primary education has been increasing in the region, around 18% of girls in Kenya do not complete primary education ^137^. Limited access to education for young girls and no avenue for adults in at-risk populations is a barrier that has a significant impact on HIV prevention.

The Ministry of Education in most countries has adopted comprehensive sexuality education (CSE) as a policy measure ^138^. This is a participatory, curriculum-based approach to equip children and young people with knowledge, skills, attitudes, and values that empower them. The curriculum covers the human body and development, sexual and reproductive health and rights, values, culture, and sexuality, relationships, gender, and diversity ^139^. In 2023, South Africa published a national HIV literacy framework to improve people’s ability to use health information rather than just understanding it, to focus on “well-informed” decisions than “appropriate” ones, and to drive a public-health perspective among populations ^140^.

## Discussion

An ecosystem lens for HIV prevention offers valuable insights into barriers and enablers at individual, interactional, and structural levels. This approach has aided policymakers and program managers in refining implementation strategies by leveraging knowledge of broader influences around a target beneficiary of HIV prevention products and services. Community-outreach initiatives, tailored behavioral interventions, comprehensive sexuality education, health system enhancements, and rights-based policy advocacy exemplify how this perspective informs program design.

The ecosystem approach has also complemented the cascade strategy that has been applied to HIV treatment and prevention ^141^. Cascades are visual interpretations of data that help monitor service access and performance by identifying barriers and inefficiencies faced by target populations and developing actionable strategies to improve programs. Cascades, whether product-specific (for condoms, PrEP, HIV testing) or population-specific (for adolescent girls and young women, sex workers, men who have sex with men), help identify drop-off points where individuals are failing to progress to the next appropriate service, and the ecosystem perspective can then help develop interventions at different levels to address these drop-off points. Thus, parsing HIV prevention into ecosystem levels is useful to tackle the implementation challenges that programs face at different levels and to develop interventions ^142^.

However, the ecosystem approach has limitations. It illuminates the factors underlying decision-making about HIV prevention – the “what”, i.e., a descriptive understanding of the current state – but does not fully explain the mechanisms driving this state over time. Without a temporal aspect, this creates a static view of the challenge. For instance, at the interaction level, availability of supportive stakeholders is an important factor, but an ecosystem lens does not explain exactly how supportive stakeholders enable or impede a user in making an HIV prevention-related decision, whether this happens at the moment of delivery or along a mental journey towards the decision. As another example, taking an ecosystem approach to designing interventions to improve an awareness program involving mass media can help understand who the important influencers or decision-makers are, what new product information users need, or what channel to leverage etc. But none of these explain the mediating mechanism causing the influence.

**Figure 1.**
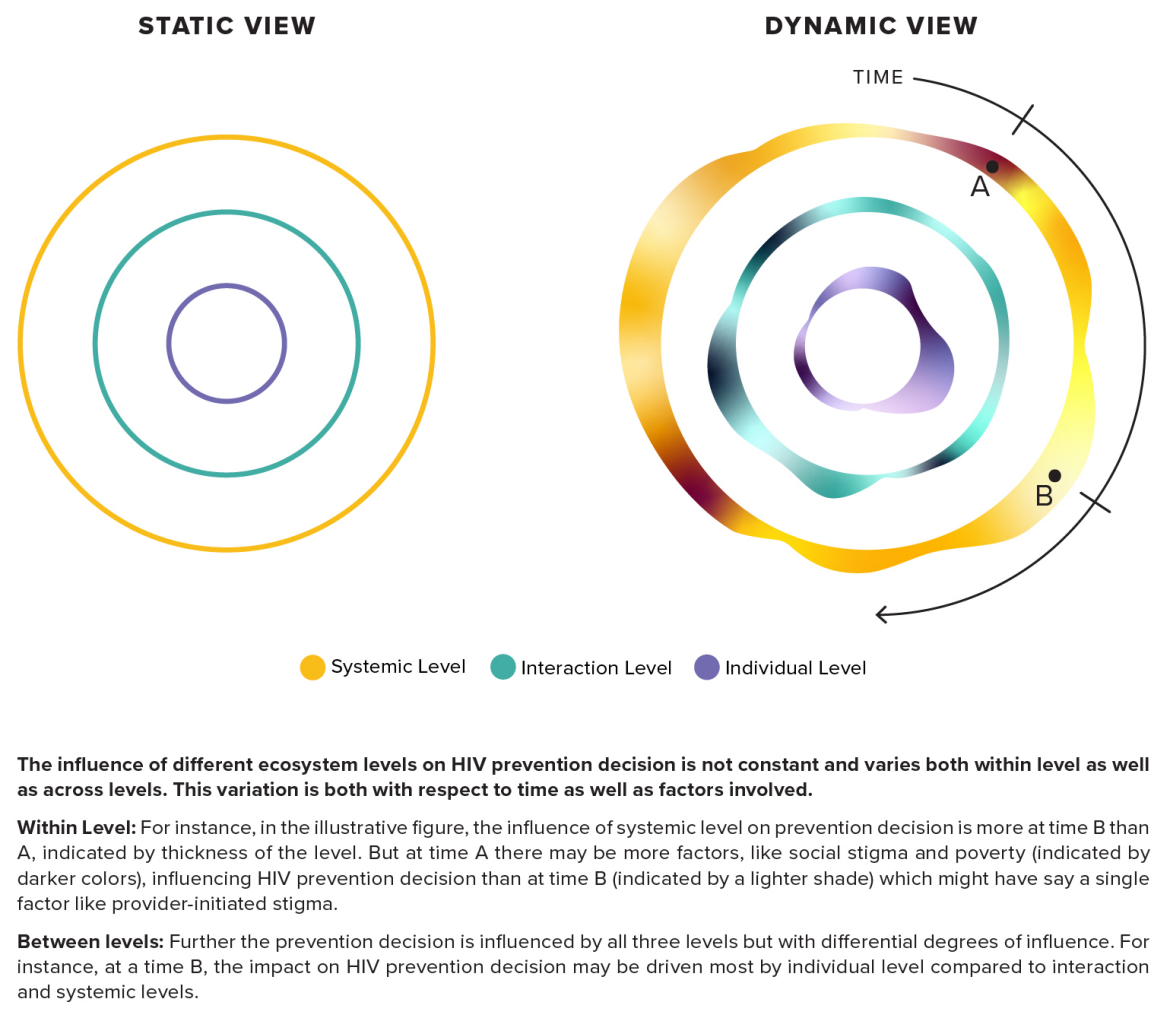
Illustrative view augmenting dynamic time-based context onto the static view of the ecosystem levels.

There is a need for an integrated approach to combine an understanding of the various factors at the different ecosystem levels with an awareness of some other interlocking and dynamic aspects of decision-making – one that internalizes the inherent dynamic context in HIV prevention decision-making and can build on changing levels of perceived risk, product availability and usage ( Figure 1).

As illustrated in Figure 1, an integrated approach can integrate the two components surrounding a HIV prevention decision – time and ecosystem level. This enables the understanding that the influence of the three ecosystem levels at any given point of time is not the same, and this influence also varies with time. Variation within levels can be understood as differing effects of the numerous factors present at that level. For instance, a systemic level can have variable influence on the HIV prevention decision if the underlying factors for the level—say, stigma and poverty—change with respect to time. Hence a policy or law criminalizing same-sex marriage at a given point in time can increase the influence of systemic level on HIV prevention decisions, as compared with other levels (individual and interactional).

Recently, the focus of HIV prevention has shifted towards self-care, defined by the World Health Organization as “the ability of individuals, families, and communities to promote health, prevent disease, maintain health, and cope with illness and disability, with or without the support of a health worker” ^142^. This paradigm shift requires moving beyond mere access and availability of HIV prevention products and services. Prevention-related decisions involve continuously varying risk perception, product availability and preferences, and desired prevention actions. Risk perception not only concerns an individual's attention but also drives the necessary intent to act. With advancements in product development, exploring users’ preferences is crucial for optimally allocating and positioning these products throughout their health-seeking journey. Traditionally, efforts have been focused on uptake and adherence—regular use of prevention products—but shifting towards effective use emphasizes selecting the right HIV prevention approach based on one’s current circumstances, integrating optimal risk assessment and positive product preference. For instance, this might mean opting for PrEP when an individual perceives themselves to be at risk of HIV and has a positive outlook towards its use, devoid of stigma or cost barriers; or deciding against PrEP when the individual assesses their risk to be negligible. An integrated socio-ecological approach, grounded in extensive user research and appreciation of these dynamic contexts, can provide policymakers and program planners with critical insights to support appropriate choices and effective use of HIV prevention products, ensuring decisions are contextually relevant and sustainable across different levels within the ecosystem.

## Conclusion

Unlike treatment, HIV prevention necessitates multi-level and multi-point interventions, lacks immediate urgency for users, demands different product usage protocols based on the perception of risk, and involves a diverse population with varying needs, risks, and vulnerabilities. As HIV-related investments continue to benefit an increasing number of individuals, leading to a decline in overall incidence and prevalence, it becomes important to develop a more comprehensive approach for public-health practitioners to extend prevention services to key and at-risk populations to limit primary transmission. An approach incorporating the dynamic, time-contingent components of decision-making can offer insights to funders and policymakers to address ineffective program implementation and drop-off from uptake of prevention products and services.

## Disclaimer

The views expressed in this article are those of the author(s). Publication in the Gates Open Journal does not imply endorsement by The Bill and Melinda Gates Foundation.

## Data Availability

No data associated with this article.
