# Supplementary material for: An Open Letter on Advancing HIV prevention: Augmenting an ecosystem-based approach to understand prevention decision-making
Source: Gates Open Res. 2025 Jan 13;8:73. Originally published 2024 Aug 2. [Version 2] doi: 10.12688/gatesopenres.16067.2 (PMC11785587; doi:10.12688/gatesopenres.16067.2)
Supplement: Peer review history

# Reviewer report: Reviewer response for version 2

**DOI:** 10.21956/gatesopenres.17699.r39346

**Published:** 2025-01-31

**Authors:** Parinita Bhattacharjee

**Affiliations:** 1 University of Manitoba, Manitoba, Canada

I approve the new version of the article

Does the article adequately reference differing views and opinions?

Partly

Are all factual statements correct, and are statements and arguments made adequately supported by citations?

Yes

Is the Open Letter written in accessible language?

Yes

Where applicable, are recommendations and next steps explained clearly for others to follow?

Partly

Is the rationale for the Open Letter provided in sufficient detail?

Yes

Reviewer Expertise:

NA

I confirm that I have read this submission and believe that I have an appropriate level of expertise to confirm that it is of an acceptable scientific standard.

# Reviewer report: Reviewer response for version 2

**DOI:** 10.21956/gatesopenres.17699.r39347

**Published:** 2025-01-17

**Authors:** Robin Schaefer

**Affiliations:** 1 Forum for Collaborative Research, University of California Berkeley, Berkeley, California, USA

I thank the authors for adequately addressing all previous feedback. I have no further feedback.

Does the article adequately reference differing views and opinions?

Yes

Are all factual statements correct, and are statements and arguments made adequately supported by citations?

Partly

Is the Open Letter written in accessible language?

Yes

Where applicable, are recommendations and next steps explained clearly for others to follow?

Yes

Is the rationale for the Open Letter provided in sufficient detail?

Partly

Reviewer Expertise:

HIV, global health, epidemiology

I confirm that I have read this submission and believe that I have an appropriate level of expertise to confirm that it is of an acceptable scientific standard.

# Reviewer report: Reviewer response for version 1

**DOI:** 10.21956/gatesopenres.17428.r37948

**Published:** 2024-10-03

**Authors:** Parinita Bhattacharjee

**Affiliations:** 1 University of Manitoba, Manitoba, Canada

This is an interesting article focusing on the demand for HIV prevention and sharing factors that may influence the uptake of HIV prevention products and services. The article prioritizes populations who are at risk of acquiring HIV. This is a timely article as we will be missing the 2025 global HIV prevention targets and have to rethink how we can increase the demand and utilization of HIV prevention products by populations that need them the most.

 However, I think the article would benefit more if the recommendations and next steps were clearer, with examples of how this framework can be used to address real-life problems. More emphasis on the application of the framework is needed. In the discussion section, the authors mention that this approach has aided policymakers and program managers in refining implementation strategies.... A few examples of such strategy revisions would be helpful.

 I also struggled to understand the dynamic time-based context and its integration into the static view of the ecosystem levels and its application. Again, the use of relevant examples to show how decision-making changes with or without the inclusion of dynamic time-based context may help.

Does the article adequately reference differing views and opinions?

Partly

Are all factual statements correct, and are statements and arguments made adequately supported by citations?

Yes

Is the Open Letter written in accessible language?

Yes

Where applicable, are recommendations and next steps explained clearly for others to follow?

Partly

Is the rationale for the Open Letter provided in sufficient detail?

Yes

Reviewer Expertise:

HIV prevention, key populations

I confirm that I have read this submission and believe that I have an appropriate level of expertise to confirm that it is of an acceptable scientific standard, however I have significant reservations, as outlined above.

# Response

**Published:** 2024-11-22

**Authors:** Nishan Gantayat

**Affiliations:** Behavioral Science, Not Applicable, Mumbai, Maharashtra, India

*Authors Response: Thank you for the feedback. We have noted this and have made changes to the paper to elaborate more on the dynamism associated with the framework with real-life HIV prevention-related decisions as examples. Also we have chosen to be specific on the strategic implications of this integrated approach by concentrating on self-care. Changes have been made to the discussion section to include these aspects.*

# Reviewer report: Reviewer response for version 1

**DOI:** 10.21956/gatesopenres.17428.r37951

**Published:** 2024-09-28

**Authors:** Robin Schaefer

**Affiliations:** 1 Forum for Collaborative Research, University of California Berkeley, Berkeley, California, USA

I thank the editors for inviting me to review this interesting article on an ecosystem-based approach to understanding HIV prevention decision-making. The authors provide a systematic overview of the range of factors influencing uptake of prevention products, at the individual, social, and structural level, and propose a dynamic framework to understand how these factors interact. The article is well-written and comprehensive. However, as noted in one of the comments below, the article needs to be situated in the broader literature on this topic and justify its added value. Below I provide further suggestions to improve the article. Once these are addressed, the article can make a useful contribution to the literature and will likely be of interest to a broad range of readers.

 Abstract

- In the second paragraph, the authors mention that they focus on “demand for HIV prevention”. I think the article is more broadly about “factors that influence uptake of prevention products”, not just demand-side factors.

Introduction

- First paragraph: This article is focused on HIV prevention, so it may be more appropriate to start the article with a focus on HIV prevention target. The 95-95-95 targets are about HIV treatment. While treatment-as-prevention is an important concept, we know that there are limits to its impact on HIV incidence, so primary prevention is most critical.
- Second paragraph: “CAB-LA” is a more commonly used acronym for long-acting injectable cabotegravir. The authors should spell this out on first occurrence. Furthermore, CAB-LA should not be described as “ART” (although it is an antiretroviral drug). Finally, the authors may want to add information on oral PrEP and nuance the statement on new PrEP products as these are not yet widely available.
- Third paragraph: Similar to the comment above, the focus should not be on an HIV treatment target (90-90-90).
- Seventh paragraph: Since the authors note that they focus on the prevention landscape in eastern and southern Africa, they may want to include some general background on the region, such as HIV incidence and HIV prevention efforts and progress (including among key populations).

Individual level

- Table 1: “Control” in the last row should also be in bold.
- Table 1: The statement in the “Relevance” section requires a reference or needs to be written in a more general sense.
- Risk saliency section, third paragraph: Here, and elsewhere, avoid language around “risky sexual behaviour”. This language can be stigmatizing. More neutral language of “behaviour associated with increased HIV acquisition risk” or something similar should be adopted.
- Coping ability section: Towards the end of this section, different PrEP products are described. This should be included in the introduction of the article.
- Coping ability section, last paragraph: This section could also mention CAB-LA, as well as other PrEP products under development (e.g., lenacapavir). Moreover, remove the word “early” when speaking of clinical trials for DVR.
- Control section, first paragraph: I think it is an overstatement to say that there is success in improving choice. While new PrEP products are becoming available, their roll-out is slow and access is limited. The choices for most people are really limited to condoms and oral PrEP.

Interaction level

- Catering to multiple needs section: This factor, as described at the beginning of this section and in the table, seems to be more about the individual rather than the interaction level. As the section goes on, it focuses more on interactions between healthcare providers and individuals, which is more suitable for this section. So the authors may want to rephrase the heading and description of this factor.
- Catering to multiple needs, second paragraph: The statement on PrEP is hugely outdated (as there has been a significant scale-up of PrEP in the region since 2020). Please revise.
- Interaction level: whole section heavily focuses on healthcare provider and the provision of health services (including through DSD models). While appropriate, there is limited focus on partners, families, and communities. Only in the “Building engaged interactions” section, there is some (limited) mentioning of partner involvement. I suggest expanding on this.
- Availability of supportive stakeholders, third paragraph: The authors may want to consider examples of peer-led services from Thailand and Vietnam.

Systemic level

- Societal constraints section: More could be included on interventions that aimed to address the harmful gender norms mentioned in this section.
- Economic vulnerabilities section: The authors combine in this section elements of individual economic vulnerabilities (first paragraph) and system constraints (second paragraph). These elements are quite different. The second aspect may deserve its own section (could be titled “Health systems constraints”).

Discussion

- Second paragraph: The authors mention cascade approaches for treatment and prevention but the included reference only relates to treatment. I suggest adding further references to the HIV prevention cascade.
- The proposed integrated approach proposed by the authors is welcomed and useful, but given that it is a key contribution of their article, they may want to expand on this (a whole article is devoted to the ‘static view’, while only one paragraph relates to the ‘dynamic view’).
- In the discussion or elsewhere, I think the article lacks a discussion on other articles that have been previously published and similarly discussed ecosystem models for HIV prevention (for instance, there is extensive literature on the socio-ecological model, which is very similar to the authors’ model). It is important to situate the article in the broader literature and justify its added value.

Conclusion

- I suggest avoiding the statement “unlike treatment” as many of the factors discussed relate to treatment as well.
- The authors mention a decline in incidence and prevalence. This may be true for southern and eastern Africa, but many other regions have been experiencing stable or even increasing incidence. The authors may want to reflect on this.

Does the article adequately reference differing views and opinions?

Yes

Are all factual statements correct, and are statements and arguments made adequately supported by citations?

Partly

Is the Open Letter written in accessible language?

Yes

Where applicable, are recommendations and next steps explained clearly for others to follow?

Yes

Is the rationale for the Open Letter provided in sufficient detail?

Partly

Reviewer Expertise:

HIV, global health, epidemiology

I confirm that I have read this submission and believe that I have an appropriate level of expertise to confirm that it is of an acceptable scientific standard, however I have significant reservations, as outlined above.

# Response

**Published:** 2024-11-22

**Authors:** Nishan Gantayat

**Affiliations:** Behavioral Science, Not Applicable, Mumbai, Maharashtra, India

Thank you for the valuable feedback. Please find below our response, which has been incorporated into the new version. 

 Abstract

- In the second paragraph, the authors mention that they focus on “demand for HIV prevention”. I think the article is more broadly about “factors that influence uptake of prevention products”, not just demand-side factors.

*Authors' Response - Noted and edits made.*

 Introduction

- First paragraph: This article is focused on HIV prevention, so it may be more appropriate to start the article with a focus on HIV prevention target. The 95-95-95 targets are about HIV treatment. While treatment-as-prevention is an important concept, we know that there are limits to its impact on HIV incidence, so primary prevention is most critical.

*Authors' Response - Noted and edits made to include prevention-relevant numbers. *

- Second paragraph: “CAB-LA” is a more commonly used acronym for long-acting injectable cabotegravir. The authors should spell this out on first occurrence. Furthermore, CAB-LA should not be described as “ART” (although it is an antiretroviral drug). Finally, the authors may want to add information on oral PrEP and nuance the statement on new PrEP products as these are not yet widely available.

*Authors' Response - Noted. Edits and additions made.*

- Third paragraph: Similar to the comment above, the focus should not be on an HIV treatment target (90-90-90).

*Authors' Response - Noted and edits made to include prevention-relevant statement. *

- Seventh paragraph: Since the authors note that they focus on the prevention landscape in eastern and southern Africa, they may want to include some general background on the region, such as HIV incidence and HIV prevention efforts and progress (including among key populations).

*Authors' Response - Noted and edits made to include region-specific numbers.*

*Individual level*

- Table 1: “Control” in the last row should also be in bold.

*Authors' Response - Noted and edits*

- Table 1: The statement in the “Relevance” section requires a reference or needs to be written in a more general sense.

*Authors' Response - Noted and edits*

- Risk saliency section, third paragraph: Here, and elsewhere, avoid language around “risky sexual behaviour”. This language can be stigmatizing. More neutral language of “behaviour associated with increased HIV acquisition risk” or something similar should be adopted.

*Authors' Response - Noted and edits to the whole of the document. Thank you for pointing this out. *

- Coping ability section: Towards the end of this section, different PrEP products are described. This should be included in the introduction of the article.

*Authors' Response - Noted. DVR and CAB-LA have already been mentioned in the introduction.*

- Coping ability section, last paragraph: This section could also mention CAB-LA, as well as other PrEP products under development (e.g., lenacapavir). Moreover, remove the word “early” when speaking of clinical trials for DVR.

*Authors' Response - Noted and edits to remove “early” and also mention CAB-LA.*

- Control section, first paragraph: I think it is an overstatement to say that there is success in improving choice. While new PrEP products are becoming available, their roll-out is slow and access is limited. The choices for most people are really limited to condoms and oral PrEP.

*Authors' Response - Noted and edits made in the language.*

 Interaction level

- Catering to multiple needs section: This factor, as described at the beginning of this section and in the table, seems to be more about the individual rather than the interaction level. As the section goes on, it focuses more on interactions between healthcare providers and individuals, which is more suitable for this section. So the authors may want to rephrase the heading and description of this factor.

*Authors' Response - Noted and edits made.*

- Catering to multiple needs, second paragraph: The statement on PrEP is hugely outdated (as there has been a significant scale-up of PrEP in the region since 2020). Please revise.

*Authors' Response - Noted and revision made*

- Interaction level: whole section heavily focuses on healthcare provider and the provision of health services (including through DSD models). While appropriate, there is limited focus on partners, families, and communities. Only in the “Building engaged interactions” section, there is some (limited) mentioning of partner involvement. I suggest expanding on this.

*Authors' Response - Noted and some edits made to expand on partner, family etc.*

- Availability of supportive stakeholders, third paragraph: The authors may want to consider examples of peer-led services from Thailand and Vietnam.

*Authors' Response - Noted and citations have been added.*

 Systemic level

- Societal constraints section: More could be included on interventions that aimed to address the harmful gender norms mentioned in this section.

*Authors' Response - Noted and some additional norms related citations have been added.*

- Economic vulnerabilities section: The authors combine in this section elements of individual economic vulnerabilities (first paragraph) and system constraints (second paragraph). These elements are quite different. The second aspect may deserve its own section (could be titled “Health systems constraints”).

*Authors' Response - Noted and edits made.*

 Discussion

- Second paragraph: The authors mention cascade approaches for treatment and prevention but the included reference only relates to treatment. I suggest adding further references to the HIV prevention cascade.

*Authors' Response - Noted and citations added.*

- The proposed integrated approach proposed by the authors is welcomed and useful, but given that it is a key contribution of their article, they may want to expand on this (a whole article is devoted to the ‘static view’, while only one paragraph relates to the ‘dynamic view’).'

*Authors' Response - Noted and have tried to expand on the section.*

- In the discussion or elsewhere, I think the article lacks a discussion on other articles that have been previously published and similarly discussed ecosystem models for HIV prevention (for instance, there is extensive literature on the socio-ecological model, which is very similar to the authors’ model). It is important to situate the article in the broader literature and justify its added value.

*Authors' Response - Noted and have tried to situate the letter in midst of other papers on socio-ecological models’ application to HIV prevention.*

 Conclusion

- I suggest avoiding the statement “unlike treatment” as many of the factors discussed relate to treatment as well.

*Authors' Response - Noted and edits made.*

- The authors mention a decline in incidence and prevalence. This may be true for southern and eastern Africa, but many other regions have been experiencing stable or even increasing incidence. The authors may want to reflect on this.

*Authors Response - Noted, and edits were made so as to be more specific to the region.*
